# A new permutation strategy of pathway-based approach for genome-wide association study

**DOI:** 10.1186/1471-2105-10-429

**Published:** 2009-12-18

**Authors:** Yan-Fang Guo, Jian Li, Yuan Chen, Li-Shu Zhang, Hong-Wen Deng

**Affiliations:** 1 School of Biomedical Engineering, Southern Medical University, Guangzhou 510515, PR China; 2 Institute of Molecular Genetics, School of Life Science and Technology, Xi'an Jiaotong University, Xi'an 710049, PR China; 3 Departments of Orthopedic Surgery and Basic Medical Sciences, University of Missouri - Kansas City, Kansas City, MO 64108, USA; 4 Center of Systematic Biomedical Research, Shanghai University of Science and Technology, Shanghai 200093, PR China; 5 College of Life Sciences and Engineering, Beijing Jiao Tong University, Beijing 100044, PR China

## Abstract

**Background:**

Recently introduced pathway-based approach is promising and advantageous to improve the efficiency of analyzing genome-wide association scan (GWAS) data to identify disease variants by jointly considering variants of the genes that belong to the same biological pathway. However, the current available pathway-based approaches for analyzing GWAS have limited power and efficiency.

**Results:**

We proposed a new and efficient permutation strategy based on SNP randomization for determining significance in pathway analysis of GWAS. The developed permutation strategy was evaluated and compared to two previously available methods, i.e. sample permutation and gene permutation, through simulation studies and a study on a real dataset. Results showed that the proposed permutation strategy is more powerful and efficient with greatly reducing the computational complexity.

**Conclusion:**

Our findings indicate the improved performance of SNP permutation and thus render pathway-based analysis of GWAS more applicable and attractive.

## Background

Genome-wide association scan (GWAS) study is becoming a popular and power method to identify genes underling complex disorders/traits [1-3]. Recent GWAS studies have discovered a number of novel genes for complex diseases, such as type 2 diabetes [4], inflammatory bowel disease [5], osteoporosis [6] and so on. However, most of current analysis methods for GWAS data were developed for analyzing individual SNPs. Simultaneously analyzing multiple SNPs/genes to detect their combined effect on phenotypes is still a challenge. Pathway analysis is an effective method that detect joint effects of SNPs or genes within a pathway in an attempt to make biologically meaningful interpretations of the GWAS data [7-12]. Moreover, pathway-based analyses of genomic data are more powerful to detect small variant effects, which may not be detectable even in very large GWAS studies.

Wang and his colleagues developed an enrichment score based pathway method for GWAS [9] by modifying the Gene Set Enrichment Analysis (GSEA) algorithm used in gene expression data [13]. In this method, genes are pre-ranked by the statistic evaluating association significance for a gene, and then an enrichment score is calculated to evaluate the concentration of genes within a pathway at the top of the entire ranked gene list of the genome. To estimate the significance of the enrichment score, permutation is a key procedure in this method [9,13]. Two permutation strategies, sample randomization and gene randomization, were then used by Wang *et al *to determine the significance of this concentration [9]. The sample randomization strategy shuffles phenotypes and re-calculates the statistic of association for each SNP and each gene in order to obtain the enrichment scores in each permutation. This permutation procedure is widely accepted as linkage disequilibrium (LD) structure among SNPs retained, however, this type of permutation is extremely time-consuming and memory-intensive as association analyses are required to be performed across the whole genome for each permutation. For gene randomization strategy, the gene statistics are shuffled and only the enrichment scores are re-calculated in each permutation. Although gene randomization can easily accomplish a large number of permutations in a short period of time, it may generate an improper null distribution of the testing statistic due to the partial usage of genome-wide association information (only the gene statistics are permuted), and thus might lead to misleading conclusion. Moreover, the performance of the two strategies can be largely inconsistent: sample randomization tends to be conservative while gene randomization yields small *p *values for most of the tested pathways. Overall, the above mentioned situations highlighted the computational challenges of the pathway-based analysis of GWAS. To the best of our knowledge, no existing study has evaluated the performance of these two permutation strategies under the situation of GWAS.

In this study, we proposed a new and efficient permutation strategy based on SNP randomization for the significance assessment in pathway-based analysis. Our approach not only dramatically reduced the computational complexity but also improved the power to detect potential pathways involving genes with joint effects on complex disorders/traits. Extensive simulations were conducted to assess the performance of the proposed strategy, the sample randomization and gene randomization strategies. We also applied the three permutation strategies to a real dataset (see [6]) for studying their relative performance. Our findings indicated that using SNP permutation can improve the performance of pathway-based GWAS.

## Methods

### Pathway-based analysis algorithm

To make this article self-contained, herein we briefly describe the pathway-based analysis algorithm that was recently extended to GWAS by Wang et al. [9]. Suppose N SNPs mapped to M genes in the whole genome have been genotyped in a sample with either population-based or family-based design. A general genome-wide association analysis has been conducted to obtain the test statistic *r*_*i *_(*i *≤ *N*; for example, *χ*^2 ^for case/control association test or *t/F *for continuous trait association test) for each SNP. Then, a statistic is constructed from SNP-level statistics to represent the statistic value for each gene, denoted as *g*_*j *_(*j *≤ *M*). Given various numbers of SNPs located in a gene with diverse LD structure among them, so far, it is not quite clear what the best strategy is to condense statistics for multiple SNPs within a gene into a single value for the gene. Following Wang et al [9], the largest absolute statistic value among all SNPs in and surrounding a gene (e.g. < 500 kb) is used to represent the statistic value of the gene, but in principle any properly combined statistic may also be used in pathway analysis [7,11,14]. For all of the M genes, we denote the sorted statistic values in a descending order as *g*_1_,..., *g*_*M*_. For any given pathway/gene set *S *consisting of *N*_*S *_genes, an enrichment score (*ES*_*S*_), which is a weighted Kolmogorov-Smirnov-like (K-S-like) running sum statistic [9,13], is calculated to reflect the overrepresentation of the genes within the set *S *at the top of the entire gene list:(1)

Where . For a given gene rank *k*, the term before the minus sign in Equation (1) evaluates the fraction of genes in S presenting up to k by weighting their association statistic, while the term behind the minus sign penalizes for the fraction of the genes not in S presenting up to k. The higher the concentration of the association signal in S at the top of the ranked gene list, the greater the value for *ES*_*S *_will be observed.

### Permutation strategies

**Figure 1:**
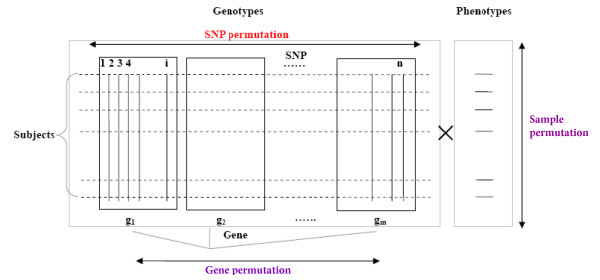
**The scheme of different permutation processes**. Horizontal dashed lines denote genome-wide genotype information of a study subject. Vertical lines denote SNP positions. Black boxes represent regions in which SNPs are annotated to a specific gene.

Permutation processes are adopted to approximate the null distribution for the test statistic of each pathway/gene set () to assess its statistical significance. Two permutation strategies, sample randomization and gene randomization, have been adopted by Wang et al. [9] However, as indicated previously, these strategies are either time-consuming or inappropriate in generating null distributions. In this study, we proposed a new permutation strategy of randomizing SNPs to assess the significance of an observed *ES*_*S *_for a given pathway S. In each permutation, this approach shuffles all SNPs across the genome and calculates the statistic for each gene. The scheme of SNP permutation process as well as the other two existing permutation processes is depicted in Fig. 1. In detail, the SNP permutation algorithm proceeds as following:

Step 1: Perform general genome-wide association analyses to determine the SNP-phenotype association statistic for every SNP in the collected dataset.

Step 2: Shuffle all SNPs across the genome to generate a permuted GWAS dataset.

Step 3: With the permuted dataset, as analyzing the observed dataset, calculate the association statistic for each gene and compute the enrichment statistic (*ES*_*S*_) for each pathway/gene set using Formula (1).

Step 4: Repeat Steps 2 and 3 till to complete a pre-set number of times (e.g. 100,000) to get the null distribution of *ES*_*s *_for each pathway/gene set.

Step 5: Based on the pool of null distributions of *ES*_*S *_over all pathways/gene sets, determine the significance of each pathway/gene set according to following strategy.

### Estimating significance

Nominal *p *value for a pathway/gene set is estimated as the fraction of permutations where *ES*_*S *_is greater than the observed one.(2)

Nominal *p *value or *ES*_*S *_may not be comparable between pathways/gene sets which usually have different number of genes. To make the enrichment score comparable between pathways, a normalized *ES*[9] is constructed based on the mean and standard deviation of , which is defined as(3)

Similar to general GWAS, multiple-testing adjustment is needed to correct the large number of pathways/gene sets tested simultaneously. False-discovery rate (FDR), a procedure frequently used to control the fraction of expected false-positive findings to stay below a certain threshold, is utilized to adjust for multiple testing and to compare the performance of the three strategies [15]. For a pathway/gene set S with , FDR (denoted as *q*_*S*_) is calculated as the ratio between the fraction of permutations over all pathways/gene sets with  and the fraction of tested pathways/gene sets with [9].(4)

### Experimental datasets

A Caucasian GWAS sample including 1,000 unrelated subjects selected from our established and expanding genetic repertoire was used for both the simulation studies and the experimental study [6]. Affymetrix Mapping 250k Nsp and Affymetrix Mapping 250k Sty arrays were applied to genotype a total of 500,568 SNPs for the 1,000 DNA samples. After quality control (detail elsewhere [6]), 312,172 SNPs relating to 14,585 genes (SNPs that are > 500 kb away from any gene were discarded, since most enhancers and repressors are < 500 kb away from genes, and most LD blocks are < 500 kb.) were retained for further exploration. SNPs mapping to multiple genes (very rare) were annotated to a single gene based on the following hierarchy: coding > intronic > 5'upstrean > 3'upstream [16]. Bone mineral density (BMD) at hip was measured for each subject.

BioCarta pathway database http://www.biocarta.com/genes/allPathways.asp was used to construct gene sets for pathway-based analysis. In total, 263 pathways annotated for humans were collected. Gene coverage for a pathway specifies the percentage of genes in a pathway which are present in the observed GWAS dataset [17]. In order to avoid misleading conclusions due to scanty representation as well as overly narrow or broad functional categories, 166 pathways with as least 85% gene coverage and containing 10-200 genes over our GWAS data were selected for following analysis.

### Simulation studies

Using our experimental genotype data, we carried out simulation studies to compare the proposed permutation strategy with sample randomization and gene randomization, based on the distribution and significance of *q*_*S *_obtained through the three permutation strategies under two scenarios.

Scenario 1: It aimed to demonstrate the differences in the distributions of *q*_*S *_for the three permutation approaches under the null hypothesis of no marker-phenotype association across the genome. It was simulated by randomly generating the phenotype data according to a standard normal distribution.

Scenario 2: It aimed to illustrate the differences in the distributions of *q*_*S *_for the three permutation approaches under the null hypothesis that there are existing gene-disease associations but no gene set enriched with genes ranking at the top of the entire gene lists in the genome. We randomly selected one gene from each of the 166 pathways. After removing duplications, seventy-five unique genes remained. Phenotype data were then simulated under the assumption that each of the 75 genes accounting for 1% genetic variation.

Before general association analyses and pathway analyses, population stratification was tested and controlled in the experimental GWAS dataset. The population stratification inflation factor *λ *for the sample (standard Pearson's chi-square test for contingency tables) [18] equaled to 1.01, suggesting that population stratification does not contribute to inflation in our studied sample. With each simulated dataset, general genome-wide association analyses were carried out by using software PLINK (version 1.05) [19]. We applied the *λ *correction to the association test statistic, which were obtained by Wald test implemented in PLINK. The adjusted statistics were then used for subsequent pathway-based analyses.

To compare the *q*_*S *_distributions, 100,000 SNP and gene permutations were conducted under both simulation scenarios and for the real dataset, respectively, but only 1000 sample permutations were performed due to the extreme computational complexity.

## Results

### Simulation studies

**Figure 2:**
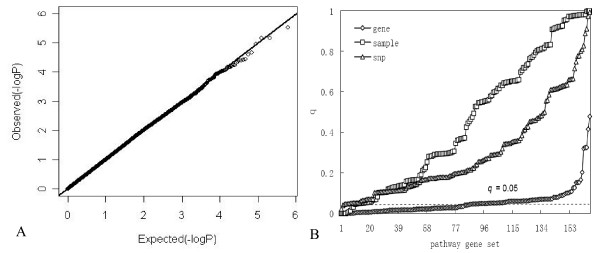
**Results of general genome-wide association analysis and pathway analysis under scenario 1**. A is quartile-quartile plot of general genome-wide association analysis. B is the *q*_*fdr *_value distribution of 166 pathways for the three permutation approaches in pathway analysis. Times of 100,000 permutations were performed for SNP or gene randomization and 1,000 permutations for sample randomization.

Fig.2 shows the *p *value quartile-quartile plot of general genome-wide association analysis and *q*_*S *_value distribution of the three permutation strategies under scenario 1. Under the null hypothesis of no marker-phenotype association, *p *values of genome-wide association were uniformly distributed and fitted the expected distribution very well (Fig. 2A). For pathway-based test (Fig. 2B), sample permutation and SNP permutation had approximately correct type I error rate, but gene permutation had an inflated type I error rate. Specifically, with a *q *value cutoff of 0.05, four (4/166*100% = 2.41%) and nine (9/166*100% = 5.42%) pathways were detected as significant by sample randomization and SNP randomization, respectively, but gene permutation claimed about sixty percent of the pathways as enriched with significant association results.

**Figure 3:**
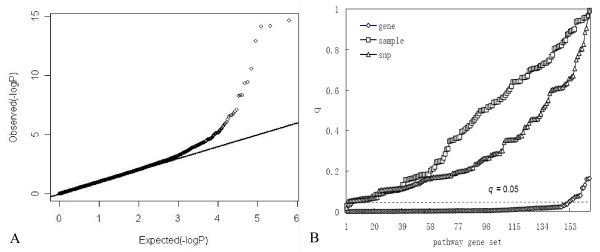
**Results of general genome-wide association analysis and pathway analysis under scenario 2**. A is quartile-quartile plot of general genome-wide association analysis. B is the *q*_*fdr *_value distributions of 166 pathways for the three permutation approaches. Times of 100,000 permutations were performed for SNP or gene randomization and 1,000 permutations for sample randomization.

Fig. 3 presents the *p *value quartile-quartile plot of general genome-wide association analysis and *q*_*S *_value distribution of the three permutation strategies under scenario 2. With simulated genetic association, we observed an excess number of SNPs in the tail of statistical distribution showing association to the phenotype (Fig. 3A). Since the genes were chosen at random to contribute to phenotype, no pathway/gene set was expected to be 'enriched' with highly significant genes and the *q*_*S *_values should be uniformly distributed. Indeed, sample permutation recognized no enriched pathway. However, the gene permutation method detected most of the pathways (91.56%) as significant with a *q*_*S *_value cutoff of 0.05. The SNP permutation approach exhibited an intermediate performance with only one *q*_*S *_value less than 0.05 (Fig. 3B).

To evaluate computation efficiency, we also assessed the CPU runtime required by the three permutation strategies in the simulation studies. Computation time as well as computation resources used in the simulation studies were summarized in Table 1. Analyses of SNP permutation and gene permutation were performed on a regular desktop computer. Considering the extreme computation intensity, only 1000 sample permutations were performed on a much more powerful cluster computer. If we run sample permutation on the same desktop computer as used for gene/SNP permutation, it took about half an hour to complete a single genome-wide association scan. Clearly, sample permutation is of extreme computational intensity, and SNP permutation is comparably time efficient as gene permutation.

**Table 1:** Runtime comparison for three permeation methods

Permutation methods	Computation resource	Times of permutation	Runtime (hour)	
			
			Scenario 1	Scenario 2
Sample	One Cluster of 4 nodes, each of which has 8 Intel^® ^Pentium^® ^P4 2.0 GHz processor, 7 GB RAM	1,000	12.32	12.35
SNP	Intel^® ^Pentium^® ^4 3.4 GHz dual processors and 2.0 GB RAM	100,000	2.81	2.81
Gene	Intel^® ^Pentium^® ^4 3.4 GHz dual processors and 2.0 GB RAM	100,000	1.90	1.89

### Application to the empirical GWAS dataset

**Figure 4:**
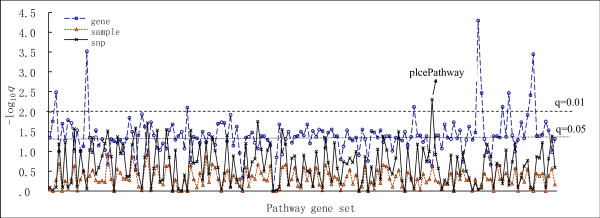
**Pathway-based genome-wide association results for the experimental dataset**. Results for randomization of gene, sample, and SNP are colored in blue, red, and black, respectively. The X-axis shows the tested pathways. The Y-axis is the log of observed *q*_*fdr *_value.

We evaluated and compared the relative performance of the study strategies by analyzing an empirical dataset, the aim of which was to explore osteoporosis susceptible genes. General genome-wide association analysis for hip BMD was conducted previously [6]. In this study, we performed the pathway-based analysis and the test results from the three permutation strategies are shown in Fig. 4. Sample permutation demonstrated very limited power as all *q*_*S *_values were greater than 0.10. While Results obtained from gene permutation showed high false error rate since more than one hundred pathways get *q*_*S *_values less than 0.05, which sharply contrast with those reported by sample permutation (correlation coefficient equals -0.16). Interestingly, signals generated by SNP permutation were analogous to those from sample permutation with similar trends and shapes but steeper peaks. The *q*_*S *_values obtained by SNP permutation were highly correlated with those obtained by sample permutation, with a correlation coefficient of 0.87 (p < 0.001). SNP permutation detected Phospholipase C-epsilon pathway (plcePathway) of the most statistically significance of enrichment after adjustment for multiple testing (*q*_*S *_≤ 0.01).

Although plcePathway is a proposed model for b2-AR- and prostanoid-receptor-mediated PLC and calcium signaling [20], its relevance to osteoporosis or bone mineral density has been reported in previous studies. Some genes in the plcePathway have been considered as important modulating factors for bone development or remodeling. For example, genetic variants of the androgen receptor may contribute to variation in bone mass as well as to predisposition to osteoporosis [21-24]. Moreover, prostanoid is reported to play an important role in the regulation of both the resorption and formation of bone [25-27].

## Discussion

Genome-wide association analysis has become a mainstay in genomic and genetic research [1,2]. Traditional strategies for GWAS have focused on identifying individual SNPs/genes that exhibit association with diseases or phenotypes. Although useful, they fail to detect biological processes that are broadly distributed across an entire network of genes which have subtle effect at the individual level [3,28]. In contrast, pathway-based analysis for GWAS, allowing researchers to consider a group of biologically related genes simultaneously, is appealing [9,13,29].

Pathway-based approach for GWAS has a number of advantages. First, pathway-based approach integrates a group of genes belonging to the same pathway/gene set in the background of the entire gene list in a genome-wide scan. Second, it preserves gene-gene correlations among specific gene sets when testing for significance. Third, pathway-based approach easily interprets a large scale association study by identifying pathways or gene set processes rather than focusing on high scoring genes, and allows researchers to refine gene subsets to elucidate biological mechanisms. Fourth, it is robust to background noises and is more likely to detect genes with moderate effects.

Permutation is a crucial process for assessing significance in pathway analysis of gene expression data [29-31], so as in pathway analysis of GWAS [9]. It is essential to develop efficient permutation schemes to facilitate applications of pathway-based GWAS. Different permutation strategies relate to different concepts of null hypothesis and give p-values with different meanings in pathway analysis of GWAS. Sample permutation assumes that the structure of genome is fixed and generates the distribution of the enrichment statistic under the assumption of no genetic effects on the disease or phenotype in question. Thus the *p *values from sample permutation mean the chance of the top hits clustering within a given pathway assuming the structure of the genome in the sample and that there are no true genetic effects. Gene permutation assumes that the risk is fixed and generates the distribution of the test statistic under the assumption that the true gene effects are randomly scattered among genes in different pathways. SNP permutation also assumes that the risk is fixed but generates the distribution of the test statistic under the assumption that the true SNP effects are randomly scattered across the genome. Thus the *p *values from both SNP permutation and gene permutation both mean that the chance of the top hits clustering within a given pathway assuming the given genetic effects but no high risk pathways. Since the null distributions are not all the same for the three permutation strategies, cautions are needed in explaining the results from pathway analyses using a specific permutation process.

Our newly proposed permutation strategy of SNP randomization is informative and efficient. On one hand, comparing to gene permutation, SNP permutation is more rational since it assumes that the existed genetic effects are randomly scattered across genome rather than among genes. In pathway analyses, the statistics for a gene are combined from SNP-level statistics. The randomization of the integrated gene statistics ignores the variation of the number of SNPs between genes. For example (please refer to Fig. 1), suppose gene A and gene B are in a gene list, where gene A consists of 10 SNPs while gene B has 20 SNPs, and *T*_*A*_, *T*_*B *_present the gene statistics for gene A and B, separately. When we shuffle the gene statistics in a permutation, gene A may take the statistic value *T*_*B*_, which is based on 20 rather than 10 SNPs. The distributions for gene statistics are expected to be different to construct from statistics of different number of SNPs. With more times of gene permutation, the number of SNPs related to the combined gene statistics for a gene from genome varies greatly, which introduces quite a lot of noises in the significance determination process. This may partly explain the inflated type I error rate of gene permutation. Since SNP permutation shuffles the SNP-level statistics and calculates gene statistic in each permutation, it overcomes the above problem in gene permutation. On the other hand, comparing to sample randomization, SNP randomization not only is highly efficient but also maintains the acceptable accuracy level (i.e. SNP randomization is not subject to an inflation of type I error rate). Although previous strategy of sample permutation is well accepted, it has not been widely applied due to its huge computation requirement to pursue a large number of replications. Given millions of genotyped markers in thousands of subjects for current GWAS, very limited replications (such as 1,000) of sample randomization can be obtained within a reasonable time frame. Overall, SNP randomization as proposed in current study inherits the merit from sample permutation making full use of the observed data and eliminates the problem of computation intensity at the same time. SNP randomization also combines the advantage of gene permutation that utilizes the output of general GWAS instead of raw genotype data. Therefore, SNP permutation is not only powerful but also cost-effective.

One potential limitation of SNP randomization might be that the independent SNP sampling may not preserve the linkage disequilibrium among SNPs and the correlation structures among functionally related genes. In our own experience, this potential problem can be overcome by increasing the number of randomization times. The larger the number of permutation, the more accurate the null distribution will be, and thus more truly reflect the distribution of enrichment of gene-phenotype association signals by random. Actually, it can be seen from the results of our empirical dataset (see Fig. 4), where *q*_*S *_values determined from SNP permutation (100,000 randomizations) is highly correlated with those from sample permutation (1,000 randomizations). Based on our application, over 50,000 SNP permutations will produce relatively stable null distribution for significance determination (The results, not shown, of 50,000, 100,000 and 150,000 SNP permutations were almost the same).

Recently, two new algorithms were proposed for pathway analysis of GWAS [7,8]. Yu *et al*. proposed one algorithm based on adaptive rank truncated product statistic to combine evidence of associations over different SNPs/genes within a pathway [7]. O'Dushlaine *et al*. proposed the other algorithm which constructs a ratio of significant SNPs to all SNPs within a pathway and compares this ratio to a distribution of ratios based on permutations [8]. Both methods employed sample permutation for assessment of the significance of tested pathways. It is possible to integrate our proposed SNP permutation strategy into their pathway analysis methods in the context of GWAS.

## Conclusion

We report here a SNP permutation scheme that is capable of effectively approximating a comprehensive null distribution to determine statistical significance, which will greatly facilitate pathway-based analysis for genome-wide data. With the improved performance and the implementation of our new SNP permutation strategy, pathway-based GWAS approach becomes more attractive and can be more broadly applied to genome-wide association datasets. Along with single marker/gene based analysis, pathway-based GWAS will enhance our understanding of pathogenesis of complex disorders.

## Authors' contributions

YG designed, conducted and analyzed the simulations and prepared a draft of this article. JL participated in project design. LZ and YC provided experimental data management and participated in project design. HD designed and coordinated the work, and participated in the interpretation of the results and the manuscript writing. All authors read and approved the final manuscript.
